# Carotenoid retention in biofortified maize using different post-harvest storage and packaging methods

**DOI:** 10.1016/j.foodchem.2017.03.158

**Published:** 2017-10-01

**Authors:** Víctor Taleon, Luke Mugode, Luisa Cabrera-Soto, Natalia Palacios-Rojas

**Affiliations:** a HarvestPlus, c/o IFPRI 2033 K Street, NW, Washington, DC 20006-1002, USA; b Department of Food Science, Postharvest Technology Research Laboratory, Stellenbosch University, Private Bag X1, Stellenbosch 7602, South Africa; c Global Maize Program, International Maize and Wheat Improvement Center (CIMMYT), CIMMYT Research Station, Km. 45 Carretera Mexico-Veracruz, El Batan, Texcoco CP 56237, Edo. de México, Mexico

**Keywords:** Biofortified maize, Carotenoid degradation, Hermetic storage

## Abstract

Orange maize is being promoted as a source of provitamin A carotenoids (pVAC) in Zambia. Carotenoid retention in orange maize grains stored in metal silos, multilayer polyethylene and common woven bags, and maize meal packaged in single and multilayer polyethylene bags was evaluated. Significant differences in total pVAC retention were found between grain storage methods (48.1–57.2%) after 6 months of storage. Total pVAC retention in hammer meal (73.1–73.5%) was higher than in breakfast meal (64.3–69.3%) after 4 months of storage; however, no differences in pVAC retention were found between meal types when stored in single and multilayer polyethylene bags. In general, β-cryptoxanthin (βCX) had higher retention than β-carotene (βC). Potential contribution of stored orange maize to the estimated average requirement of children and women was 26.5% and 24.3%, respectively. Orange maize meal can provide significant amounts of provitamin A to diets of Zambians even after 4 months of storage.

## Highlights

• Degradation rate of βCX was 51% lower than βC during storage of orange maize grain.
• Grain storage methods with 16% oxygen level reduced carotenoid degradation by 9.1%.
• Orange maize is an alternative to improve vitamin A status of deficient populations.

## 1 Introduction

About 54% of children 0.5–5 years old in Zambia are vitamin A deficient (WHO, 2009). Maize is the most important crop for human consumption in Zambia, where annual production is 2.8 million metric tons and per capita intake is 287 g per day among women and 172 g per day among children 2–5 years old (FAOSTAT, 2016, Hotz et al., 2011). Maize consumed in Zambia as well as in other Southern and Eastern African countries is mostly white-grained, and the consumption of yellow grains is minor (Smale et al., 2015). White maize has low levels of total carotenoids and does not have provitamin A carotenoids (pVAC), while common yellow maize has up to 66 µg g^−1^ of total carotenoids but typically has less than 2 µg g^−1^ of pVAC, making it a poor source of provitamin A (Pixley et al., 2013). The first group of biofortified orange maize varieties, which has been conventionally bred as an alternative to improve vitamin A status of populations, contains 6–9 µg g^−1^ of pVAC. Biofortified orange maize varieties have been grown commercially in Zambia, Nigeria and Ghana since 2013 (Alamu et al., 2015, Pixley et al., 2013, Smale et al., 2015). Malawi, Zimbabwe and Tanzania have also released biofortified orange maize recently (Hwang et al., 2015, International Maize and Wheat Improvement Center (CIMMYT), 2016).

The efficacy of biofortified orange maize as an alternative for improving vitamin A status in children has been shown by two studies conducted in Zambia (Gannon et al., 2014, Palmer et al., 2016). The current target for maize biofortification efforts is 15 µg pVAC per g of maize, which should be sufficient for children and pregnant women to reach 50% of the estimated average requirement (EAR), assuming several factors including up to 50% pVAC losses during grain storage and cooking (Lividini and Fiedler, 2015, Saltzman et al., 2013). However, recent modeling studies on the adoption of pVAC maize and its benefits in addressing vitamin A deficiency (VAD) in Zambia suggest that positive impact may be limited by both the low retention of carotenoid during storage and postharvest grain losses (Lividini & Fiedler, 2015).

Postharvest grain losses of up to 30% occur in African countries when maize is stored using common storage methods, such as artisanal silos or woven bags. Losses are mainly attributed to infestation with insects, rodents or fungi and accumulation of mycotoxins (Tefera et al., 2011). Improved methods for preventing losses during storage have been promoted in African countries, including the use of multilayer polyethylene bags (PICS) and metal silos hermetically sealed with or without a candle (Tefera et al., 2011, Yakubu et al., 2011). Respiration of insects and fungi reduces oxygen (O_2_) and increases carbon dioxide (CO_2_) available in hermetic storage systems, creating a modified atmosphere that prevents migration of moisture and growth of such (micro) organisms (Baoua et al., 2012, Garcia-Lara et al., 2013).

In Zambia, maize is consumed mostly as *nshima*, a stiff porridge prepared with maize meal and boiling water (Smale et al., 2015). Different types of maize meal are used to prepare *nshima*, namely hammer meal, breakfast meal and roller meal. Hammer and breakfast meals are the most consumed throughout the country. Hammer meal is made of whole maize grain ground in a hammer or stone mill, whereas breakfast meal is produced by grinding the maize endosperm after removing most of the pericarp and germ. Typically, hammer and breakfast meals are not consumed on the same day they are milled but are stored for 4–6 months in woven bags or single layer polyethylene bags. The shelf life of hammer meal is shorter than that of breakfast meal due to the high fat content in the germ that leads to rancidity (Fiedler et al., 2013, Gwirtz and Garcia-Casal, 2014).

It is known that carotenoids in different food matrices are highly degraded by oxygen, temperature and light during storage (De Moura et al., 2015, Rodriguez-Amaya, 2015). In matrixes with high exposure to oxygen, such as flour, it was found that the oxygen level had greater influence on carotenoid degradation than temperature (Alves et al., 2012, Bechoff et al., 2010). In general, pVAC degradation in biofortified maize grain may reach 60% when grain is stored for 6 months using traditional methods, depending on the genotype (De Moura et al., 2015, Ortiz et al., 2016). Also, previous studies reported 78–100% pVAC retention when cooking freshly ground biofortified orange maize meal to prepare *nshima* (Gannon et al., 2014, Mugode et al., 2014). However, to our knowledge, there is no information on carotenoid retention during storage of breakfast and hammer meal produced with biofortified orange maize.

The objectives of this study were to: 1) determine the rate of carotenoid degradation during kernel storage under different storage conditions; 2) assess pVAC retention in hammer and breakfast meal stored for 4 months using different packaging methods; and 3) estimate the percent EAR of vitamin A provided by *nshima* made with biofortified orange maize.

## 2 Materials and methods

### 2.1 Maize grain for grain storage methods

Maize grains of biofortified orange maize variety GV664A collected from three commercial farms in Central Province, Zambia, in June 2015, were used to evaluate different grain storage methods. Ears were harvested at <14% grain moisture, dried for three days in shaded and ambient temperature conditions and then shelled using a mechanical sheller. Grains were immediately placed in different containers, as described in the grain storage methods section.

### 2.2 Grain storage methods

Maize grains from Central Province were stored for 6 months under ambient conditions with a daily average temperature of 22 °C. Average daily temperature during the first 60 days of storage was 17 °C, with a weekly variation between 15 and 19 °C. Grains were stored in traditional 50-kg woven bags, 100-kg PICS bags, 100-kg metal silos with candle and 100-kg metal silos without candle. Fifty kilograms of grain were used for each storage condition, in triplicate. PICS bags were filled and hermetically closed, as described by Baoua et al. (2012). Metal silos, with and without candle, were hermetically sealed with rubber bands, as described by Tefera et al. (2011). In addition, 50 kg of unshelled grain in woven bags were stored under the same conditions. Grains stored at the same temperature and for the same time period but in 3.75-kg vacuum-sealed aluminum Mylar bags were used as controls. Oxygen absorbers were placed inside the aluminum Mylar bags and immediately heat-sealed to create the vacuum. Total available O_2_ at the beginning of the experiment was 27.9 L for metal silos and 5.2 L for woven and PICS bags. Grain samples for carotenoid analysis were taken at 0, 30, 90 and 180 days of storage from the metal silos and PICS, whereas for the woven bags, samples were taken at 0, 5, 10, 15, 30, 60, 90, 135 and 180 days of storage. For carotenoid analysis, samples from each repetition were collected in triplicate. Immediately after sampling, samples were placed in aluminum Mylar bags with oxygen absorbers and stored at −20 °C until shipment and analysis.

### 2.3 Evaluation of milling and packaging types

To evaluate different packaging types for maize meal, orange maize grains of variety GV664A were collected from Lusaka Province, Zambia, (June 2015) but not from the Central Province, due to logistical reasons. Samples from three different commercial farms were used. After shelling the ears, grains were stored for 3 months in 50-kg woven bags following common storage practices used by households in this region of Zambia. After storage, grains were processed either as breakfast meal or hammer meal. Breakfast meal was obtained by removing about 10% of the germ and pericarp from 150 kg of maize grains (90% extraction rate) using a de-huller and then milling the remaining endosperm into coarse flour using a commercial hammer mill and a sieve with 0.6-mm openings. Hammer meal was obtained by grinding 150 kg of whole maize kernels using the same commercial hammer mill and sieve.

### 2.4 Packaging types for maize meal

Hammer and breakfast meal were stored in 100-kg multilayer PICS and in 3.75-kg polyethylene and aluminum bags for 4 months under ambient conditions. Daily average temperature during the storage period was 24 °C, with a monthly variation between 23 and 25 °C. The amount of maize meal used was 25 kg for PICS bags and 2.5 kg for polyethylene and aluminum bags, in triplicate. Total available O_2_ at the beginning of hammer meal storage was 2.92 L for PICS bags and 0.28 L for polyethylene bags. For breakfast meal, total available oxygen was 3.58 L for PICS bags and 0.34 L for polyethylene bags. Availability of O_2_ in aluminum bags with oxygen absorbers for hammer and breakfast meal was <0.02 L. Maize meal samples for carotenoid analysis were taken at 0, 30, 60, 90 and 120 days of storage. Samples were stored at −20 °C in aluminum Mylar bags with oxygen absorbers until shipping and analysis. Sampling for carotenoid analysis was done in triplicate for each repetition.

### 2.5 Oxygen measurement

The O_2_ and CO_2_ levels were monitored with a portable gas analyzer Checkmate 3 (PBI Dansensor, Ringstead, Denmark) by inserting a needle through a rubber septum placed in each storage unit. Before the experiments, small 5-mm holes were opened in the metal silos and double septa were placed in the hole to measure oxygen over time without interfering with the atmosphere generated inside the silos during the storage period. Double septa were also placed in PICS bags for the same purpose. For grains, O_2_ and CO_2_ were measured daily for the first 90 days in modified metal silos, every week in PICS bags and just before sample collection in woven bags. For maize meal, O_2_ and CO_2_ were measured before sample collection.

### 2.6 Physical characterization of maize grain and meal

Bulk density of maize kernels and flour was determined using AACC Method 55-10 (AACC, 2000). Kernel dimensions were measured using a digital caliper (Salinas & Vazquez, 2006). The true density of grain and maize meal was determined using a liquid displacement method and particle size was measured as described by Salinas and Vazquez (2006).

### 2.7 Carotenoid analysis

All reagents and chemicals used were of HPLC and analytical grade. All *trans*-β-carotene (βC), 9-*cis*-β-carotene (9-*cis*-βC), 13-*cis*-β-carotene (13-*cis*-βC), β-cryptoxanthin (βCX), lutein (LUT) and zeaxanthin (ZEA) were purchased from CaroteneNature (Ostermundigen, Switzerland). An internal standard of β-apo-8′-carotenal was purchased from Sigma-Aldrich (St. Louis, MO). Methyl *tert*-butyl ether, tetrahydrofuran, dichloroethane, methanol, acetonitrile, ethyl alcohol and potassium hydroxide were obtained from Merck (Darmstadt, Germany). Carotenoid extraction and analysis was done as described in Rosales, Agama-Acevedo, Bello-Perez, Gutiérrez-Dorado, and Palacios-Rojas (2016). Total pVAC was computed as βC + (1/2)(13-*cis*-βC) + (1/2)(9-*cis*-βC) + (1/2)(βCX), which also represents total β-carotene equivalents (βCE).

### 2.8 Rate of carotenoid degradation and calculation of carotenoid retention

Rate of carotenoid degradation (*k*) during storage was calculated as:

$$\mathrm{Ln}\frac{x}{x_{0}}=-\mathrm{kt}$$

where *x* is the concentration of carotenoid at time *t* (day), *x*_0_ is the concentration of carotenoid at the beginning of storage, and *k* is the constant rate of carotenoid degradation (day^−1^). Since the experiment was carried out under field conditions, the temperatures were not constant. However, in order to use this rate of degradation (*k*) model, which assumes constant temperatures, a permissible temperature variation of 4 °C (weekly variation of daily average) for grains and 3 °C (monthly variation of daily average) for maize meal were used. With such restrictions, the storage time used for calculating the carotenoid degradation rate in grains was 60 days, and 120 days in maize meal.

The true retention (%TR) was calculated as:

$$\%\text{TR}=\frac{\text{pVAC per g of maize after storage}\;(\text{dry basis})*100}{\text{pVAC per g of maize before storage}(\text{dry basis})}$$

### 2.9 Percent vitamin A EAR from orange maize nshima

To estimate the potential contribution of orange maize *nshima* to% vitamin A EAR for children 4–6 years old and women in Zambia (under typical grain and maize meal storage conditions), two scenarios of maize intake were considered based on assumptions from modeling studies by Lividini and Fiedler (2015). The first scenario assumed that biofortified maize fully substituted consumption of white maize and that the daily maize intake was 287 g for women and 172 g for children 4–6 years old. The second scenario assumed partial adoption of biofortified maize; therefore, consumption was 117 g for women and 71 g for children 4–6 years old. To calculate total true retention during storage of maize grain and meal, a maximum of 8 months of storage as grain and 4 months of storage either as hammer or breakfast meal was assumed. These values were based on the common shelf life of hammer meal which is 4 months (Gwirtz & Garcia-Casal, 2014). The *k* values obtained (day^−1^) for maize grains and meal using traditional storage and packaging methods were used to calculate the monthly carotenoid retention for grains and hammer meal and combined to generate the total retention for a season in Zambia. Vitamin A EAR of 500 µg for women and 275 µg for children 4–6 years was used (IOM, 2001). Retention during cooking of *nshima* was considered to be 78%, as reported by Gannon et al. (2014).

### 2.10 Statistical analysis

Data were expressed as mean ± standard deviation (SD). For the experiments with grains, the effects of storage methods and time were evaluated using a 2-factor ANOVA model. ANOVA and linear regression analysis were performed using the MIXED and REG procedures in SAS 9.4. Means separation was calculated using Duncan’s Multiple Range Test. Differences between means were considered significant at *p* < 0.05.

## 3 Results and discussion

### 3.1 Physical characteristics of GV664A maize grains and meal

Grain samples from Central Province had 11% moisture content before milling. The thousand-kernel weight was 224 ± 2 g. Average kernel dimensions were 8.91 ± 0.25 mm length, 8.14 ± 0.14 mm width and 4.08 ± 0.21 mm thickness. True density was 1.288 ± 0.030 kg L^−1^ and bulk density was 0.794 ± 0.010 kg L^−1^. Bulk density was lower than values reported by Ortiz et al. (2016) for flint type orange maize (0.887–0.984 kg L^−1^ at 8–16% moisture), but much higher than common white or yellow maize (0.682–0.772 kg L^−1^ at 14% moisture) (Sahasrabudhe, 2015). High bulk density compared to the values reported for common yellow maize varieties was possibly due to the high true density of grains as well as small grain dimensions. A higher bulk density could be important because when the storage unit is hermetically sealed, less O_2_ will be available inside to react with carotenoids, compared to most common commercial white and yellow maize.

Bulk density of breakfast and hammer meal was 0.842 ± 0.005 and 0.764 ± 0.003 kg L^−1^, respectively. True density for both meal types was 1.50 ± 0.01 kg L^−1^. Particle size of breakfast meal was finer than hammer meal, with 25% and 46% of particles bigger than 0.50 mm and 14% and 5% smaller than 0.25 mm, respectively. Significantly higher bulk density in breakfast meal could be attributed to factors such as different particle size distribution and less germ content compared to hammer meal.

### 3.2 Oxygen content in storage units

The gas composition in the woven and PICS bags at the beginning of the experiment was that of normal air (20.6% O_2_ + 0.03% CO_2_), whereas for metal silos with lit candles, the O_2_ level was 16.5%. Initial O_2_ concentration in metal silos with lit candles was lower than in the other storage methods, due to the O_2_ consumed by the candles inside the silos. To our knowledge, no reports of the minimum O_2_ level in a hermetically sealed metal silo with a candle inside are available. The average O_2_ level observed in the aluminum silos during the 90 days of monitoring in grain was 18.5%, whereas in PICS bags it was 18.7%. The oxygen level in both hermetic storage methods did not drop to less than 1–5% as suggested by other studies that monitored O_2_ levels in hermetic jars in the laboratory and PICS bags in the field. This difference might be explained because in such studies accelerated O_2_ consumption inside the storage methods used was due to the growth of inoculated insects (Moreno-Martinez et al., 2000, Mutungi et al., 2015, Tefera et al., 2011, Yakubu et al., 2011). Natural O_2_ reduction due to insect respiration in hermetically sealed commercial metal silos filled with grain has not been clearly documented; however, in field experiments insect infestation was reduced to only 4% when using small commercial metal silos (De Groote et al., 2013).

### 3.3 Carotenoid composition of maize grains and hammer meal

Total carotenoid content of orange maize grains from the Central Province before being stored as grain was 20.4 ± 0.2 µg g^−1^ in dry basis. Individual pVAC levels were between 1.2 ± 0.1 and 4.9 ± 0.4 µg g^−1^, representing 55.1% of the total carotenoids quantified and 7.6 ± 0.1 µg g^−1^ pVAC (Table 1). The most predominant pVAC found was βCX with 24.1%, followed by βC with 19.1%. The 9-*cis*-βC and 13-*cis*-βC isomers were found in lower proportions, with 6.1% and 5.8%, respectively. The proportions of βCX and βC were within the ranges observed in other orange maize genotypes, 19.0–28.0% for βCX and 12–23% for βC (Mugode et al., 2014, Muzhingi et al., 2016, Ortiz et al., 2016, Pillay et al., 2014). Non pVAC ZEA and LUT represented 31.2% and 12.2% of the total quantified carotenoids, respectively (Table 1).

**Table 1:** Carotenoid content (µg g^−1^ dry weight)ɑ of orange maize grain, meal and bran variety GV664A grown in Zambia.

Grain source	Grain fraction or type	LUT	ZEA	βCX	13-*cis*-βC	βC	9-*cis*-βC	pVAC
Central Province§	Whole grain	2.6 ± 0.2^a^	6.5 ± 0.1^a^	4.9 ± 0.4^a^	1.2 ± 0.0^a^	3.9 ± 0.2^a^	1.2 ± 0.1^a^	7.6 ± 0.1^a^
Lusaka Province€	Whole grain	1.7 ± 0.1^b^	4.1 ± 0.2^b^	3.4 ± 0.3^b^	0.7 ± 0.0^c^	2.1 ± 0.1^c^	0.9 ± 0.0^b^	4.6 ± 0.3^c^
Lusaka Province€	Breakfast meal	2.0 ± 0.3^b^	4.9 ± 0.8^b^	3.6 ± 0.5^b^	0.9 ± 0.0^b^	2.6 ± 0.2^b^	1.1 ± 0.1^a^	5.4 ± 0.1^b^
Lusaka Province€	Bran + germ	0.3 ± 0.2^c^	0.8 ± 0.4^c^	0.7 ± 0.2^c^	0.3 ± 0.1^d^	0.6 ± 0.2^d^	0.3 ± 0.1^c^	1.3 ± 0.4^d^

ɑ Different lower case letters indicate significant differences within columns (*p* < 0.05). Values are average ± standard deviation (three farms). LUT = lutein; ZEA = zeaxanthin; βCX = β-cryptoxanthin; 13-*cis*-βC = 13-*cis*-β-carotene; βC = all *trans*-β-carotene; 9-*cis*-βC = 9-*cis*-β-carotene; pVAC = provitamin A carotenoids (expressed as β-carotene equivalents). Whole grain = hammer meal.

§ Freshly harvested grains from Central Province.

€ Obtained from grain stored for 3 months in farms.

Considering that maize grain used to produce either hammer meal or breakfast meal is usually not fresh, and to understand carotenoid degradation in maize meal stored under common storage conditions in Zambia, orange maize harvested in Lusaka Province was stored for three months and then milled to produce these two meal types. Total carotenoid content in the stored grains used to produce both meal types was 12.8 ± 0.7 µg g^−1^, whereas their pVAC was 4.6 ± 0.3 µg g^−1^, representing 55.0% of the total carotenoids identified (Table 1). The most predominant pVAC was βCX with 26.4%, whereas βC, 9-*cis*-βC, 13-*cis*-βC represented 16.0%, 6.9% and 5.7%, respectively. These stored grains from Lusaka Province had 37% less pVAC than fresh grains from Central Province. Taking into consideration the storage time and environmental effect, the difference in pVAC between maize grains from the two provinces was reasonable and representative of what could be expected in typical farms in Zambia. The carotenoid content in hammer meal was the same as the carotenoid content found in its unprocessed stored grains, since the particle size reduction required to analyze carotenoids in maize grains resembles the production of hammer meal.

### 3.4 Carotenoid profile of breakfast meal

In freshly milled breakfast meal at 90% extraction rate obtained with the stored grain from Lusaka Province, the total carotenoid content and pVAC was 14.8 ± 1.1 µg g^−1^ and 5.4 ± 0.1 µg g^−1^, respectively. The pVAC present in the highest proportion was βCX (24.4%), followed by βC (17.6%), 9-*cis*-βC (6.9%) and 13-*cis*-βC (5.8%), whereas ZEA and LUT represented 32.1% and 13.3% of total quantified carotenoids. In general, the proportions of carotenoids found in breakfast meal were similar to what was found in their grains. Levels of LUT, ZEA and βCX were not significantly different between freshly milled breakfast meal and hammer meal; however, βC, 9-*cis*-βC, 13-*cis*-βC and pVAC were significantly higher in breakfast meal (Table 1).

The bran fraction obtained when producing breakfast meal had the lowest total carotenoid and pVAC with 3.0 ± 1.2 and 1.3 ± 0.4 µg g^−1^, respectively (Table 1). Proportions of βCX, βC, 9-*cis*-βC and 13-*cis*-βC were 23.6%, 21.2%, 11.7% and 11.2%, respectively. The lower carotenoid content found in the bran is in agreement with reported values for other maize genotypes (Burt et al., 2010, Ndolo and Beta, 2013). The low carotenoid content in bran removed from grain when producing breakfast meal may explain the higher carotenoids levels observed in freshly milled breakfast meal compared to hammer meal. However, the total proportion of pVAC removed from the bran represented only 3% of the pVAC present in the kernel, whereas the proportion of bran removed was 10%. This is an important aspect to consider when calculating per capita pVAC intake based on total maize grain consumed at the household level, because elimination of germ and pericarp from breakfast meal (mostly endosperm) likely results in greater carotenoid intake compared to hammer meal.

### 3.5 Carotenoid degradation in grain under different storage conditions

**Fig. 1:**
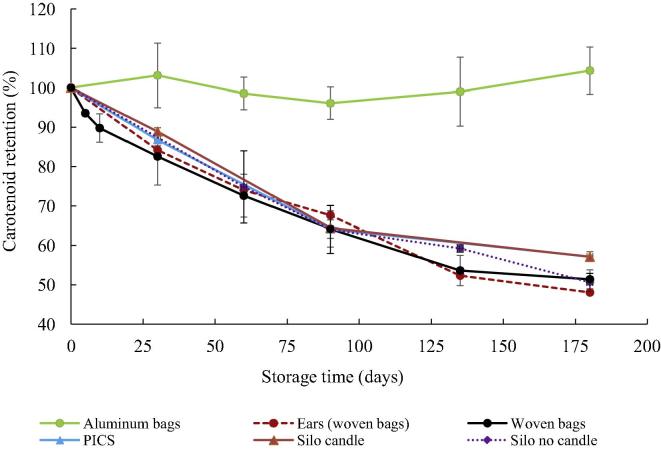
Effect of storage method on carotenoid pVAC retention in orange maize grain variety GV664A stored using different methods for 180 days under ambient conditions in Zambia.

The pVAC contents in maize grains after 180 days of storage were 8.0 ± 0.4, 4.3 ± 0.2, 4.3 ± 0.3, 3.9 ± 0.2, 3.8 ± 0.2 and 3.6 ± 0.1 µg g^−1^, representing true retention of 104.3%, 57.2%, 57.1%, 51.4%, 50.6% and 48.1% for aluminum bags, PICS bags, silo with candle, woven bags, silo without candle and ears in woven bags, respectively (Fig. 1). Carotenoid retention in maize stored in aluminum bags with oxygen absorbers was the highest, followed by silos with candles and PICS bags, whereas grains in woven bags, silos without candles and ears in woven bags had the lowest carotenoid retention (*p* < 0.05). The minimum retention values found in the different storage methods used were slightly higher than the 40.0% retention limit reported by Mugode et al. (2014) for orange maize stored at 20 °C (average temperature) using traditional methods in Zambia. However, in that study, fast degradation was observed during the first two weeks of storage and no information on the kinetics of degradation was provided.

Degradation rate of βCX, 13-*cis*-βC, βC and 9-*cis*-βC in orange maize grains stored in woven bags was calculated for the first 60 days of storage. Based on the coefficient of determination (*r*^2^) observed when obtaining the degradation rate constant values, all pVAC followed first-order degradation kinetics. First-order degradation has been also reported for pVAC in orange maize grains (Ortiz et al., 2016) and for βC in sweet potato and cassava (Bechoff et al., 2010, Bechoff et al., 2015). When comparing *k* values of each carotenoid, βC had the highest *k* values (0.00777 ± 0.00060), followed by 13-*cis*-βC (0.00511 ± 0.00051) and βCX (0.00381 ± 0.00058), whereas 9-*cis*-βC (0.00276 ± 0.00043) had the lowest *k* values (*p* < 0.05). The *k* values for βCX and βC were within the ranges reported for different orange maize genotypes by Ortiz et al. (2016), who also reported higher *k* values for βC than βCX in maize stored at 22 °C under non-hermetic conditions. The lower *k* values (51% lower) observed for βCX compared to βC are relevant because maize grain bred with higher proportions of βCX, as opposed to βC, could have better pVAC retention during storage and consequently higher impact on vitamin A deficient populations. Higher βCX stability during storage together with higher βCX bioavailability compared to βC, and βCX’s similar bioconversion and bioefficacy to βC (Schmaelzle et al., 2014), adds strong support to the βCX breeding strategy proposed by Dhliwayo, Palacios-Rojas, Crossa, and Pixley (2014), which is based on genetically controlling pVAC accumulation in maize grains. Large genetic diversity for βCX content in maize is already documented and makes the strategy feasible (Swarno, Palacios-Rojas, Kaeppler, & Babu, 2015).

In general, these results suggest that improved storage methods that reduce grain losses (such as hermetic silos with low O_2_ and PICS bags) could also contribute to reduce pVAC degradation in orange maize grains. The lower degradation in these storage systems may be due to the lower oxygen content inside them. Despite the very high carotenoid retention in aluminum bags with oxygen absorbers, this method is too expensive and not feasible for use in rural households; however, results showed that oxygen is a key factor when storing orange maize grains at room temperature in Zambia.

Grains to be stored must be free of insect contamination; however, given the poor management practices in the field and during harvesting and drying, usually larvae or even adult insects are present in the grain. Baoua et al. (2012) and Garcia-Lara et al. (2013) reported that when the grain is naturally infested with insects before storage in hermetic silos, available oxygen inside the silos decreases to 5% due to insect respiration, resulting in minimum grain damage at the end of the storage period. If under such conditions grain integrity is well preserved, there is a possibility that the carotenoid degradation rate during storage of initially infested grains will be lower than in non-infested grains.

### 3.6 Degradation kinetics of carotenoids in maize meal

**Fig. 2:**
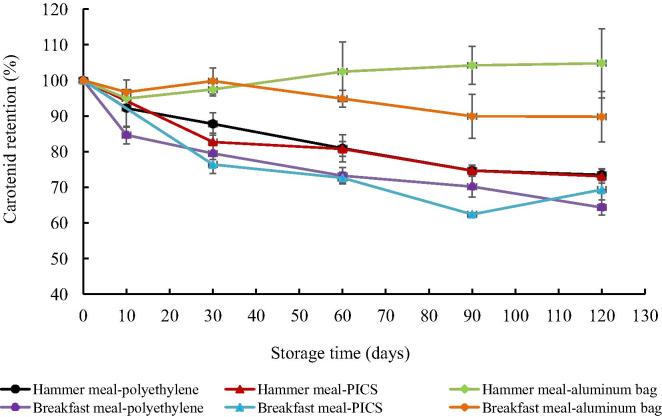
Effect of storage method on pVAC retention in breakfast meal and hammer meal made from orange maize variety GV664A and stored using different packaging methods for 120 days under ambient conditions in Zambia.

Carotenoid levels in maize meal after storage were significantly lower than in the freshly milled maize for all the packaging methods used except aluminum bags. The pVAC levels at the end of the storage period were 4.8 ± 0.5, 3.3 ± 0.2, and 3.3 ± 0.2 µg g^−1^ for hammer meal in aluminum, PICS and polyethylene bags, whereas for breakfast meal these values were 4.9 ± 0.3, 3.8 ± 0.3 and 3.5 ± 0.1 µg g^−1^, respectively. The retention of pVAC in hammer meal after 120 days of storage was 104.8%, 73.5%, 73.1% for aluminum bags, polyethylene bags and PICS, whereas for breakfast meal it was 89.8%, 64.3% and 69.3%, respectively (Fig. 2). As observed in the grain, carotenoid retention in maize meal stored in aluminum bags was the highest among all storage methods after 120 days of storage (*p* < 0.05).

Degradation constant rates for pVAC were determined for breakfast and hammer meal stored in different packaging methods. All pVAC followed first-order degradation kinetics for all packaging methods based on the high coefficient of determination (*r*^2^ > 0.8), except for the control (aluminum foil with oxygen absorbers), which had low *r*^2^ values (Table 2). Hammer meal in single layer polyethylene bags had lower *k* values than breakfast meal in the same packaging type for 13-*cis*-βC, 9-*cis*-βC, βC and pVAC, whereas hammer meal in PICS bags had lower *k* values than breakfast meal in PICS bags for 13-*cis*-βC, βC and pVAC (Table 2).

**Table 2:** Degradation constant rate (*k*)^a^ expressed as day^−1^ for pVAC in orange maize meal stored for 120 days in different packaging methods.

Type of meal	Package	Carotenoid	*k*	*r*^2^	*p*
BF meal	Polyethylene	βCX	0.00327^a^	0.92	<0.01
BF meal	PICS		0.00349^a^	0.82	<0.01
BF meal	Aluminum		0.00088^b^	0.26	0.16
HM meal	Polyethylene		0.00283^a^	0.93	<0.01
HM meal	PICS		0.00292^a^	0.92	<0.01
HM meal	Aluminum		0.00023^b^	−0.05	0.99

BF meal	Polyethylene	13-*cis*-βC	0.00345^a^	0.89	<0.01
BF meal	PICS		0.00323^a^	0.89	<0.01
BF meal	Aluminum		0.00107^c^	0.77	<0.01
HM meal	Polyethylene		0.00215^b^	0.97	<0.01
HM meal	PICS		0.00215^b^	0.95	<0.01
HM meal	Aluminum		0.00059^c^	0.67	<0.01

BF meal	Polyethylene	βC	0.00526^a^	0.88	<0.01
BF meal	PICS		0.00508^a^	0.88	<0.01
BF meal	Aluminum		0.00141^c^	0.70	<0.01
HM meal	Polyethylene		0.00344^b^	0.95	<0.01
HM meal	PICS		0.00344^b^	0.95	<0.01
HM meal	Aluminum		0.00072^c^	0.37	<0.01

BF meal	Polyethylene	9-*cis*-βC	0.00327^a^	0.92	<0.01
BF meal	PICS		0.00300^ab^	0.91	<0.01
BF meal	Aluminum		0.00095^c^	0.75	<0.01
HM meal	Polyethylene		0.00221^b^	0.97	<0.01
HM meal	PICS		0.00232^b^	0.96	<0.01
HM meal	Aluminum		0.00043^c^	0.36	0.43

BF meal	Polyethylene	pVAC	0.00416^a^	0.91	<0.01
BF meal	PICS		0.00416^a^	0.88	<0.01
BF meal	Aluminum		0.00095^c^	0.52	<0.01
HM meal	Polyethylene		0.00296^b^	0.95	<0.01
HM meal	PICS		0.00302^b^	0.94	<0.01
HM meal	Aluminum		0.00035^c^	0.14	0.15

ɑ Different lower case letters indicate significant differences within columns (*p* < 0.05). Values of *k* are average of three farms. BF = breakfast meal; HM = hammer meal; βCX = β-cryptoxanthin; 13-*cis*-βC = 13-*cis*-β-carotene; βC = all *trans*-β-carotene; 9-*cis*-βC = 9-*cis*-β-carotene; pVAC = provitamin A carotenoids (expressed as β-carotene equivalents).

Significant differences in *k* values for all carotenoids were observed between packaging types after 120 days of storage. Similar to the results on grains, the *k* values of βC (0.00344–0.00526) in maize meal were significantly higher (*p* < 0.05) than for all other pVAC (0.00215–0.00349), suggesting that βC is more unstable than all other pVAC in maize regardless of the physical properties of the matrix. These *k* values for βC are lower than values reported in sweet potato flour (0.00930–0.01930) when stored at 20–30 °C (Bechoff et al., 2010), and dry cassava granules stored at 19–33 °C (*k* = 0.0083–0.02710) (Bechoff et al., 2015). Considering the large effect of oxygen observed in grains and maize meal, it could be hypothesized that an important reason why *k* values obtained for maize meal were lower than reported values for sweet potato at slightly lower temperature (20 °C) is that maize meal granules are bigger than sweet potato flour granules. The dense matrix of maize meal granules could also have restricted the reaction of oxygen from the environment with the carotenoids compared to cassava granules (at 20 °C) which have light granules. Similarly, the higher *k* values of breakfast meal compared to hammer meal may be due in part to the smaller particle size of breakfast meal which exposes it to oxygen, but also, to the presence of tocochromanols (mainly tocopherols) in hammer meal. The germ contained in hammer meal could have prevented degradation of its carotenoids, considering that tocopherols are present mostly in the corn germ, and tocopherols reduce the degradation of carotenoids due to their antioxidant capacity (Muzhingi et al., 2016). The integrity of the cellular compartments may be crucial for reducing the oxygen exposure of carotenoids, thereby their stability. Similar observations were reported by Rosales et al. (2016) when comparing pVAC retention during traditional and extruded lime-cooking process. Retention of pVAC was higher in traditional methods where grains are milled after being cooked, as compared to methods where milling occurs before extrusion.

In general, based on the calculated *k* values for maize grain and hammer meal of variety GV664A, and assuming a maximum storage period of 8 months for grain and 4 months for meal, the average retention during storage using traditional methods was 46.7% for hammer meal and 43.8% for breakfast meal. Therefore, biofortified orange maize should be commercialized as grain and milled shortly before consumption, preferably as hammer meal. Also, farmers and consumers should be advised to consume biofortified orange maize first, before extensive carotenoid degradation occurs, and consume white maize when orange maize is no longer available. For urban commercialization of milled orange maize, the time it is kept on store shelves should be minimized.

### 3.7 Contribution of orange maize nshima to daily intake of vitamin A

Assuming total replacement of white maize with GV664A or similar cultivars of biofortified orange maize (containing about 7.6 µg g^−1^ pVAC) and 78% retention during cooking, *nshima* prepared with orange maize hammer meal made from freshly ground maize provides 56.8% and 52.1% EAR, while maize stored for up to 4 months provides 26.5% and 24.3% EAR, for children 4–6 years old and women, respectively. Biofortified orange maize provides more than the target 50% EAR if grain and flour are processed fresh, with no storage period. New orange maize genotypes currently in the variety release pipeline contain 15 or more µg g^−1^ pVAC, and the% EAR that they provide will increase correspondingly (www.cimmyt.org). Given the genotypic differences in pVAC retention, further research is needed to verify that new cultivars with 15 or more µg g^−1^ indeed provide the predicted 112.6% and 103.4% EAR when consuming fresh maize and 52.6% and 48.2% EAR for stored maize, for children 4–6 years of age and women, respectively.

Considering Lividini and Fiedler’s (2015) estimates that orange maize will be only partially adopted, with consumption levels reaching 71 g for children 4–6 years of age and 117 g for women, then the expected contribution of orange maize to% EAR would be 21.7% and 19.7%, respectively, when storing grain and meal of varieties with 15 µg g^−1^ pVAC. These values are nutritionally important although they are lower than the target 50% EAR expected for biofortified maize in Zambia. Under this scenario, alternatives to ensure higher retention would be important, as well as continued breeding for higher content of pVAC, including higher βCX content. Breeding for increased content of antioxidant compounds (i.e., tocochromanols) mainly in the grain endosperm could also enhance resistance to oxidative degradation of pVAC (Muzhingi et al., 2016). To reduce vitamin A deficiency in Zambia, nutritional education, diet diversification, sanitation and disease prevention efforts, as well as creating awareness and demand for biofortified orange maize should be continued.

## 4 Conclusion

Carotenoid degradation was similar for ears and shelled orange maize. Improved maize storage methods, such as PICS bags and metal silos with low O_2_, enhance carotenoid retention, compared to traditional storage methods. More emphasis should be given to increase βCX in new orange maize genotypes because βCX is more stable than βC in all storage and packaging methods for either grains or maize meal. Also, pVAC carotenoids are more stable in hammer meal compared to breakfast meal, mainly due to the larger particle size and smaller surface exposed to oxygen for hammer meal relative to breakfast meal. The potential scenario that had the lowest pVAC in cooked nshima, considering the current pVAC levels of orange maize being cultivated in Zambia, provided 26.5% and 24.3% of vitamin A% EAR for children 4–6 years old and women. The time during which orange maize meal sits on market shelves should be minimized so that its contribution to the vitamin A status of Zambians is maximized.
